# Safety and Efficacy of Adding Fixed-Combination Brinzolamide/Timolol Maleate to Prostaglandin Therapy for Treatment of Ocular Hypertension or Glaucoma

**DOI:** 10.1155/2015/131970

**Published:** 2015-10-01

**Authors:** Anton Hommer, Douglas A. Hubatsch, Juan Cano-Parra

**Affiliations:** ^1^Private Office, Vienna, Austria; ^2^Alcon Laboratories, Inc., Fort Worth, TX 76134, USA; ^3^Hospital Municipal de Badalona, 08911 Barcelona, Spain

## Abstract

*Purpose*. To evaluate the safety and efficacy of adding brinzolamide 1%/timolol maleate 0.5% fixed combination (BTFC) to a prostaglandin analog (PGA). *Methods*. This was a 12-week, open-label, single-arm study of patients with open-angle glaucoma or ocular hypertension with intraocular pressure (IOP) not sufficiently controlled after ≥4 weeks of PGA monotherapy. The primary outcome was mean IOP change from baseline at week 12. Other outcomes included IOP change from baseline at week 4, percentage of patients achieving IOP ≤18 mmHg at week 12, and patient experience survey responses at week 12. *Results*. Forty-seven patients were enrolled and received treatment. The most commonly used PGAs were latanoprost (47%) and travoprost (32%). Mean ± SD IOP was decreased at week 12 (17.2 ± 4.1 mmHg) compared with baseline (23.1 ± 3.0 mmHg; *P* < 0.001, paired *t*-test); IOP at week 4 was 17.2 ± 3.3 mmHg. At week 12, 70% of patients achieved IOP ≤18 mmHg. Patient-reported symptoms (e.g., pain and redness) were mostly unchanged from baseline. Twenty-eight adverse events (AEs) were reported; the most frequently reported AE was headache (3 events in 2 patients). *Conclusion*. Adjunctive BTFC + PGA therapy was effective and well tolerated. IOP decreased by 6 mmHg at weeks 4 and 12.

## 1. Introduction

Elevated intraocular pressure (IOP) is the major risk factor for development of primary open-angle glaucoma (POAG), and higher IOP levels are associated with increased risk of glaucoma-related blindness [1, 2]. Lowering IOP with pharmacologic therapy reduces the rate of disease progression (e.g., optic neuropathy) and visual field loss and reduces the risk of conversion from ocular hypertension (OH) to glaucoma [2–4]. Pharmacotherapy is usually initiated with a single ocular hypotensive agent [2], and prostaglandin analogs (PGAs) or *β*-blockers are frequently prescribed as initial monotherapy because of their IOP-lowering efficacy and safety profiles [5].

However, many patients require multiple IOP-lowering agents to achieve or maintain sufficient IOP reduction [4]. Combining glaucoma medications with complementary mechanisms of action may further reduce IOP. PGAs (e.g., latanoprost, travoprost, and bimatoprost) reduce IOP by increasing uveoscleral and, to a lesser extent, trabecular aqueous humor outflow, whereas *β*-blockers (e.g., timolol) and carbonic anhydrase inhibitors (brinzolamide, dorzolamide) decrease aqueous humor production [2]. Increasing the number of individual medications that patients must self-administer increases treatment complexity and may reduce adherence to glaucoma medication regimens [6, 7]. For patients with IOP insufficiently controlled with a single medication, adding a fixed-combination adjunctive therapy to their monotherapy provides additive IOP-lowering efficacy with only 2 medication bottles (versus 3 with individual agents). Glaucoma treatment guidelines typically suggest stepwise addition of 1 ocular hypotensive medication at a time for patients who require additional IOP reduction [8–10]; however, adding a fixed-combination glaucoma medication to monotherapy has been reported to be well tolerated and effective [11].

Brinzolamide 1%/timolol maleate 0.5% fixed-combination ophthalmic suspension (BTFC; AZARGA, Alcon Laboratories, Inc., Fort Worth, TX) has been demonstrated to effectively lower IOP in patients with POAG or OH, including those transitioned because of insufficient reduction in IOP with previous therapy [12].

The purpose of this study was to evaluate the efficacy and safety of adding BTFC to PGA monotherapy in patients with POAG or OH who were responsive to but inadequately controlled by their PGA monotherapy.

## 2. Methods

### 2.1. Study Design and Treatment

This was a 12-week, prospective, interventional, single-arm, open-label study conducted at 5 sites in Austria and Spain from March 2011 to April 2013 (registration identifiers: ClinicalTrials.gov, NCT01263444; EudraCT, 2010-022948-21). The study consisted of 3 visits: a screening/baseline visit and follow-up visits conducted after 4 weeks (±3 days) and 12 weeks (±3 days) of treatment. Follow-up visits were scheduled for approximately the same time of day as the baseline visit (±1 hour). At the conclusion of the baseline visit, patients were instructed to continue their PGA therapy and to self-administer 1 drop of BTFC (10 mg/mL [1.0%] brinzolamide/5 mg/mL [0.5%] timolol) into the study eye(s) twice daily at 8 AM and 8 PM for 12 weeks. The 8 PM dose was instilled at a 5-minute interval from the once-daily PGA dose. For eyes not qualifying for inclusion in the study, IOP was required to be controlled either with no pharmacologic intervention or with prostaglandin monotherapy. Patients using contact lenses during the study were instructed to remove lenses for instillation of study medication and to wait ≥15 minutes after instillation before reinsertion.

This study was approved by the Ethikkommission der Stadt Wien (Austria) and CEIC Fundación Oftalmológica del Mediterráneo (Spain) and was performed in compliance with the ethical principles of the Declaration of Helsinki and Good Clinical Practice. Before screening, patients provided written informed consent using an ethics board-approved consent form.

### 2.2. Patients

Eligible patients were aged ≥18 years with an existing clinical diagnosis of OH, POAG, or pigment dispersion glaucoma in both eyes. Additional inclusion criteria were IOP responsive to but insufficiently controlled by PGA monotherapy after ≥4 weeks of treatment before screening; baseline IOP (on PGA therapy) ≥20 mmHg in at least 1 eye (the study eye) and ≤35 mmHg in both eyes; and best-corrected visual acuity (BCVA) of 6/60 Snellen (1.0log⁡MAR) or better in each eye.

Key exclusion criteria were medical history of allergy, hypersensitivity, or poor tolerance to any components of the study medications; any primary or secondary glaucoma other than POAG, OH, or pigment dispersion glaucoma; narrow angle with complete or partial closure in either eye; progressive retinal or optic nerve disease other than glaucoma; corneal dystrophies or concurrent conjunctivitis, keratitis, or uveitis in either eye; history or risk of uveitis or cystoid macular edema; history of herpes simplex; any abnormality in the study eye preventing reliable applanation tonometry or fundus/anterior chamber examination; intraocular conventional or laser surgery <3 months before screening; any cardiac or pulmonary condition that precluded safe administration of a topical *β*-blocker; any use of corticosteroids ≤30 days before the study or during the study; use of any carbonic anhydrase inhibitor; severely impaired renal function; hyperchloremic acidosis; myasthenia gravis; and participation in any other investigational study ≤30 days before baseline. Women who were pregnant, lactating, or of childbearing potential and not using a reliable method of birth control were also excluded. For patients using systemic medications that may affect IOP (e.g., oral *β*-blockers, *α*-agonists and blockers, angiotensin-converting enzyme inhibitors, and calcium channel blockers), a stable course was required for ≥7 days before baseline and throughout the study.

### 2.3. Efficacy Outcomes and Assessments

The primary efficacy outcome was the mean change in IOP from baseline, when patients were receiving PGA monotherapy, to week 12, when patients were receiving BTFC plus PGA. Other assessments included the mean change in IOP from baseline to week 4, percentage of patients reaching the target IOP of ≤18 mmHg at week 12, and mean change in patient experience survey responses from baseline to week 12. IOP measurements were performed at baseline, week 4, and week 12 by Goldmann applanation tonometry; tonometers were calibrated before patient screening was initiated, and IOP measurements for individual patients were performed by the same operator using the same tonometer at all visits. The patient experience survey was administered at the baseline visit and at week 12. Symptom severity was defined as minimal (symptom present but barely noticeable), mild (symptom definitely present but does not limit activity), moderate (symptom present and severe enough to partially limit activity), or severe (symptom present and is incapacitating).

### 2.4. Safety Outcomes and Assessments

Safety was assessed by monitoring adverse event (AE) reports. Ocular signs and BCVA at weeks 4 and 12 were also assessed. Ocular signs were assessed in both eyes at each study visit by slit-lamp biomicroscopy of the eyelids, conjunctiva, cornea, iris, anterior chamber, and lens. Findings were graded as 0.5 (trace), 1 (mild), 2 (moderate), or 3 (severe). BCVA was measured using a Snellen visual acuity chart at each study visit; if >1 error occurred on a given line, values were rounded up.

### 2.5. Statistical Analyses

Efficacy outcomes were analyzed in the intent-to-treat (ITT) population (i.e., all patients who received study medication and had at least 1 on-therapy study visit) and in the per-protocol (PP) population (i.e., all patients who received study medication, completed all study visits, and had no major protocol deviations) using data from the study eye. Safety outcomes were analyzed using data for all patients who received study medication.

Mean IOP change from baseline measured at week 12 was analyzed by 2-sided paired *t*-tests; results at week 4 were considered supportive data. Changes in patient experience survey responses were evaluated by 1-way analysis of variance. Demographic information, percentages of patients with IOP ≤18 mmHg versus >18 mmHg, and safety data were summarized descriptively. Statistical analyses were performed using SAS (SAS Institute, Cary, NC, USA) by an independent biostatistician; *P* < 0.05 was considered statistically significant.

A power calculation determined that completion of the study by ≥40 patients was sufficient to detect a difference in mean IOP ≥1.5 mmHg (week 12 versus baseline; SD = 2.8 mmHg) with 90% power. To ensure that ≥40 patients completed the study, the target enrollment was 50 patients.

## 3. Results

### 3.1. Patients

Forty-seven patients were enrolled and included in the safety and ITT data sets; 38 patients completed the study. Most patients were aged ≥66 years (72.3%, *n* = 34/47), and approximately half were female (51.1%, *n* = 24/47). Patient diagnoses at enrollment were OH, 66.0% (*n* = 31); POAG, 55.3% (*n* = 26); and pigment dispersion glaucoma, 2.1% (*n* = 1). Two diagnoses were reported for some patients, causing total diagnoses to exceed 100%. Latanoprost was the most frequently used PGA therapy (46.8%, *n* = 22/47), followed by travoprost (31.9%, *n* = 15/47), bimatoprost (17.0%, *n* = 8/47), and tafluprost (4.3%, *n* = 2/47). Nine patients discontinued from the study; 8 discontinuations were because of AEs, and 1 patient withdrew consent. One patient was excluded from the PP data set (*n* = 37) because of a protocol deviation (i.e., exclusion criteria: corneal dystrophy).

### 3.2. Efficacy Outcomes

**Figure 1:**
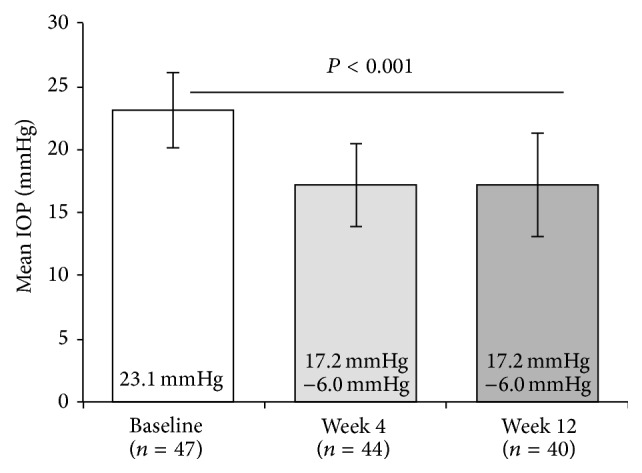
IOP reduction from baseline at weeks 4 and 12. Bars represent mean IOP ± SD; mean IOP reduction from baseline is indicated inside bars. IOP = intraocular pressure. Baseline versus week 12, *P* < 0.001; 1-way analysis of variance with a post hoc, 2-sided paired *t*-test.

**Figure 2:**
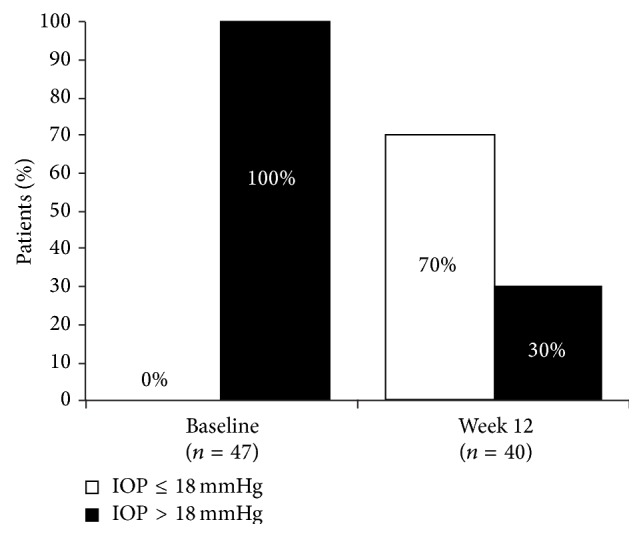
Percentage of patients with IOP ≤18 mmHg and >18 mmHg at baseline and week 12. Patient percentages are indicated inside bars. IOP = intraocular pressure.

Efficacy data were similar in the ITT and PP data sets; results for the ITT population are presented. IOP (mean ± SD) was 23.1 ± 3.0 mmHg at baseline (*n* = 47; range, 20.0–32.0 mmHg), 17.2 ± 3.3 mmHg at week 4 (range, 10.0–25.0 mmHg), and 17.2 ± 4.1 mmHg at week 12 (*n* = 40; range, 10.0–28.0 mmHg). The overall mean ± SD IOP reduction from baseline was 6.0 ± 3.2 mmHg at week 12 (*P* < 0.001); similar results were observed at week 4 (Figure 1). Analysis by PGA type for travoprost and latanoprost verified that the decrease from baseline at week 12 was significant for both (5.1 ± 3.4 mmHg and 7.1 ± 2.9 mmHg, resp.; *P* < 0.001 for both). At week 12, 70% of patients achieved the target IOP of ≤18 mmHg. At baseline, no patients had IOP ≤18 mmHg (Figure 2).

There were no significant differences between baseline and week 12 in the number of patients who reported experiencing a symptom or event on the patient experience survey (Table 1). At week 12, there was a nonsignificant decrease from baseline in pain severity in or around the eyes when exposed to light (*P* = 0.072). Among patients who reported stinging or burning (baseline, *n* = 14/47; week 12, *n* = 12/39), there was a difference in symptom severity between baseline and week 12 (*P* = 0.035). At baseline, 21.4% of patients reported severity of this symptom as “minimal,” 64.3% as “mild,” and 14.3% as “moderate.” At week 12, 53.9%, 15.4% and 30.8% of patients reported minimal, mild, and moderate severity, respectively.

### 3.3. Safety Outcomes

A total of 28 AEs were reported by 21 patients (Table 2); 16 AEs (57.1%) were determined to be related or possibly related to the study medication. One serious AE (moderate pseudostenocardia related to study medication) occurred and led to study discontinuation. Seven additional patients discontinued because of AEs (allergic conjunctivitis; tiredness and insomnia; rhinitis sicca; headache, metallic taste, ocular foreign body sensation, blurred vision, and 1 unspecified AE; stomachache; headache; and eye pain). Nearly all AEs were mild or moderate in severity (96.4%, *n* = 27/28), and 96.4% of AEs resolved by the end of the study. The most frequently reported AE was headache (3 events reported for 2 patients).

Slit-lamp observations were similar among visits. At baseline, observations for eyelids, conjunctiva, cornea, iris, anterior chamber, and fundus were normal for most patients (57.4% to 100.0%); abnormalities were reported as “trace” or “mild” for most eyes. Examination of the lens at baseline was abnormal for most patients (72.3%); however, most abnormalities were reported as “trace” or “mild.” BCVA was unchanged from baseline to week 4 or week 12.

## 4. Discussion

Reducing IOP to minimize disease progression is the standard of care for glaucoma and OH. Several studies have demonstrated that maintaining sufficiently low IOP may slow or prevent progression of visual field defects. For many patients, long-term monotherapy does not maintain target IOP, and many patients benefit from a combination of 3 ocular hypotensive agents [4, 12]. The goal of this study was to evaluate the safety and efficacy of adding adjunctive BTFC in patients with open-angle glaucoma or OH who had insufficient IOP reduction with PGA monotherapy alone.

At baseline, when patients were receiving only PGA monotherapy, mean IOP was 23.1 mmHg. After 12 weeks of adjunctive BTFC therapy, mean IOP decreased by 6.0 mmHg to 17.2 mmHg. This reduction was observed at week 4 and was maintained through study completion. IOP was >18 mmHg in all patients at baseline, but at week 12, 70% of patients achieved the target IOP of ≤18 mmHg. The most common AE was headache, which was reported by 2 patients, and nearly all AEs were mild or moderate in severity. BCVA and slit-lamp biomicroscopy observations were unchanged from baseline throughout the study.

Maintaining IOP levels ≤18 mmHg may decrease the risk of glaucoma progression. A meta-analysis of 5 retrospective studies of patients with POAG or exfoliative glaucoma with ≥5 years of follow-up demonstrated that glaucoma progressed in 51% of patients with IOP >18 mmHg, whereas 78% of patients with mean IOP of 18 mmHg did not progress [3]. In general, as mean IOP increased above 18 mmHg, the percentage of patients who remained stable decreased; likewise, at mean IOP levels below 18 mmHg, the percentage of patients who remained stable increased [3]. In the current study, only 30% of patients failed to achieve IOP ≤18 mmHg after 12 weeks of BTFC adjunctive to a PGA, which was a marked improvement from baseline. Rates of visual field decline have been shown to decrease with even small reductions in IOP [13, 14]. The Early Manifest Glaucoma Trial demonstrated that for every 1 mmHg decrease in IOP, the progression risk decreases by as much as 10% [14]. These studies suggest that the additive IOP-lowering efficacy of BTFC adjunctive to PGA therapy described in the current study may decrease risk of glaucoma progression by reducing IOP in patients not sufficiently controlled with PGA therapy alone.

Our findings are in agreement with previous reports describing increased IOP-lowering efficacy of 3-medication combinations that included a PGA, a carbonic anhydrase inhibitor, and a *β*-blocker [15–17]. In the current study, most patients were receiving latanoprost or travoprost at enrollment; adding adjunctive BTFC reduced mean IOP by an additional 6 mmHg from levels achieved with the PGA monotherapy. Previously, the stepwise IOP-lowering efficacy of travoprost monotherapy, fixed-combination travoprost/timolol, and fixed-combination travoprost/timolol plus brinzolamide was assessed in a single-arm, open-label study of patients with POAG or OH [16]. After a washout period, travoprost monotherapy decreased mean diurnal IOP by 6.2 mmHg; fixed-combination travoprost/timolol reduced IOP by an additional 3.1 mmHg, and adding brinzolamide to the fixed combination further reduced IOP by 1.9 mmHg [16]. Mean diurnal IOP with travoprost/timolol plus adjunctive brinzolamide was 5.0 mmHg lower than levels achieved with travoprost monotherapy [16]. An observer-masked, placebo-controlled crossover comparison of patients with open-angle glaucoma responsive to but insufficiently controlled with latanoprost monotherapy demonstrated that fixed-combination dorzolamide/timolol adjunctive to latanoprost reduced 24-hour mean IOP by 5.6 mmHg from levels that were maintained with latanoprost alone [17]. Similar 3-medication combinations have also shown increased IOP-lowering efficacy compared with fixed combinations of 2 ocular hypotensive agents; mean IOP reductions with travoprost/timolol plus adjunctive brinzolamide or dorzolamide/timolol plus adjunctive latanoprost ranged from 1.9 mmHg to 5.2 mmHg compared with the fixed combinations alone [15, 18].

The AEs observed in this study were consistent with the known side effects of BTFC and topical PGAs [19, 20]. Nearly all AEs were mild in severity, and the most frequently reported AE was headache. Most ocular symptoms and events reported by patients were similar at baseline and week 12; there was a nonsignificant improvement in pain severity in or around eyes during light exposure at week 12. No changes from baseline in slit-lamp observations or BCVA were observed.

Potential limitations of this study include the single-arm, open-label design and 12-week study duration. Patients' baseline IOP may have been influenced by noncompliance with the prior medication, and adherence to study medication may have been increased because of participation in a clinical trial. Future studies with multiarm or crossover designs and longer follow-up durations would be valuable.

The use of different PGAs in this study was intentional, as it was meant to reflect clinical practice where a variety of PGAs are used based on the patient's profile and the physician's preference. Although not powered to show differences between groups, analysis by PGA type (travoprost and latanoprost) verified that IOP decreased significantly for both treatment groups when BTFC was added. However, if a specific PGA and brinzolamide/timolol combination were not to lower IOP, the effect would be masked because the mean change in IOP was calculated across all PGA types.

In conclusion, BTFC adjunctive therapy reduced IOP when added to a PGA in patients with OH or POAG whose IOP was poorly controlled with PGA monotherapy alone. IOP reductions were evident after 4 weeks of combined BTFC plus PGA therapy and were maintained through 12 weeks. BTFC was well tolerated, and the ocular AEs reported were consistent with the current BTFC safety profile. Adding adjunctive BTFC therapy may provide a safe and effective option for patients requiring additional IOP reduction beyond that provided by a PGA.

## Figures and Tables

**Table 1:** Patient experience survey data.

	Incidence and severity	Patients, *n* (%)^*∗*^		*P* value
		Baseline *n* = 47	Week 12 *n* = 39	
Do you experience or have you noticed		At this moment	Immediately following instillation of study medication	

Pain in or around your eyes when exposed to light?	Yes	5 (10.6)	5 (12.8)	0.753^†^
	Minimal	0	1 (20.0)	0.072^‡^
	Mild	0	1 (20.0)	
	Moderate	1 (20.0)	3 (60.0)	
	Severe	4 (80.0)	0	
Blurred or dim vision?	Yes	9 (19.2)	14 (35.9)	0.081^†^
	Minimal	5 (55.6)	6 (42.9)	0.733^‡^
	Mild	2 (22.2)	3 (21.4)	
	Moderate	2 (22.2)	5 (35.7)	
	Severe	0	0	
Stinging or burning?	Yes	14 (29.8)	12 (30.8)	0.921^†^
	Minimal	3 (21.4)	7 (53.9)	0.035^‡^
	Mild	9 (64.3)	2 (15.4)	
	Moderate	2 (14.3)	4 (30.8)	
	Severe	0	0	
A feeling that something is in your eyes or under your lids?	Yes	11 (23.4)	11 (28.2)	0.611^†^
	Minimal	2 (18.2)	4 (40.0)	0.385^‡^
	Mild	4 (36.4)	4 (40.0)	
	Moderate	5 (45.5)	2 (20.0)	
	Severe	0	0	
Deep pain in or around your eyes?	Yes	3 (6.4)	3 (7.7)	0.812^†^
	Minimal	0	1 (33.3)	0.368^‡^
	Mild	1 (33.3)	0	
	Moderate	2 (66.7)	2 (66.7)	
	Severe	0	0	
Redness in your eyes^§^?	Yes	13 (27.7)	11 (28.2)	0.955^†^
	Minimal	5 (38.5)	3 (27.3)	0.366^‡^
	Mild	4 (30.8)	7 (63.6)	
	Moderate	3 (23.1)	1 (9.1)	
	Severe	1 (7.7)	0	

^*∗*^For each question, the percentage of “yes” responses was calculated based on the group size, and severity data were calculated as the percentage of patients who responded “yes.”

^†^Numbers of “yes” responses at week 12 versus baseline were analyzed by 1-way analysis of variance.

^‡^Symptom severity responses at week 12 versus baseline were analyzed by 1-way analysis of variance.

^§^Causes of redness were not specified.

**Table 2:** Adverse events (safety population).

	Episodes, *n* (%) *n* = 28
Patients experiencing ≥1 adverse event, *n* (%)^*∗*^	21 (44.7)
Adverse event severity	
Mild	23 (82.1)
Moderate	4 (14.3)
Severe	1 (3.6)
Adverse event	
Headache	3 (10.7)
Allergic conjunctivitis	1 (3.6)
Allergic rhinitis	1 (3.6)
Ankle pain	1 (3.6)
Burning/eyelid swelling	1 (3.6)
Blurred vision	1 (3.6)
Conjunctival discomfort	1 (3.6)
Corneal superficial keratitis	1 (3.6)
Crusting of lashes	1 (3.6)
Dry eye	1 (3.6)
Eye pain	1 (3.6)
Lid erythema	1 (3.6)
Metallic taste	1 (3.6)
Ocular foreign body sensation	1 (3.6)
Pseudostenocardia^†^	1 (3.6)
Punctate keratopathy	1 (3.6)
Rhinitis sicca	1 (3.6)
Scheduled knee total endoprosthesis due to gonarthrosis	1 (3.6)
Stomachache	1 (3.6)
Subjective poorer vision	1 (3.6)
Tinnitus	1 (3.6)
Tiredness and insomnia	1 (3.6)
Trace keratitis	1 (3.6)
Upper respiratory infection	1 (3.6)
Worsening of dorsal pain	1 (3.6)
Unknown^‡^	1 (3.6)

^*∗*^Calculated as the percentage of patients in the safety population (*n* = 47).

^†^Serious adverse event.

^‡^A description was not available for 1 event in 1 patient.
